# Integrated multi-omics analyses combined with western blotting discovered that cis-TSG alleviated liver injury via modulating lipid metabolism

**DOI:** 10.3389/fphar.2024.1485035

**Published:** 2024-11-20

**Authors:** Tekleab Teka, Jiang Wu, Patrick Kwabena Oduro, Ze Li, Chenxi Wang, Hao Chen, Lin Zhang, Haitao Wang, Liming Wang, Lifeng Han

**Affiliations:** ^1^ State Key Laboratory of Component-based Chinese Medicine, Tianjin Key Laboratory of TCM Chemistry and Analysis, Tianjin University of Traditional Chinese Medicine, Tianjin, China; ^2^ School of Pharmacy, Wollo University, Dessie, Ethiopia; ^3^ Shenzhen Technology University, Shenzhen, China; ^4^ Jacobs School of Medicine and Biomedical Sciences, The State University of New York, University at Buffalo, Buffalo, NY, United States; ^5^ Haihe Laboratory of Modern Chinese Medicine, Tianjin, China

**Keywords:** *cis*-TSG, stilbene glycoside, cholestasis, metabolomics, lipidomics, transcriptomics

## Abstract

**Background:** *Polygonum multiflorum* shows dual hepatoprotective and hepatotoxic effects. The bioactive components responsible for these effects are unknown. This study investigates whether *cis*-2,3,5,4'-tetrahydroxystilbene-2-O-*β*-D-glucoside (*cis*-TSG), a stilbene glycoside, has hepatoprotective and/or hepatotoxic effects in a liver injury model.

**Methods:** C57BL/6J mice were administered *α*-naphthylisothiocyanate (ANIT) to induce cholestasis, followed by treatment with cis-TSG. Hepatoprotective and hepatotoxic effects were assessed using serum biomarkers, liver histology, and metabolomic and lipidomic profiling. Transcriptomic analysis were conducted to explore gene expression changes associated with lipid and bile acid metabolism, inflammation, and oxidative stress.

**Results and Discussion:** ANIT administration caused significant liver injury, evident from elevated alanine aminotransferase (ALT) and aspartate aminotransferase (AST) levels and dysregulated lipid metabolism. *cis*-TSG treatment markedly reduced ALT and AST levels, normalized lipid profiles, and ameliorated liver damage, as seen histologically. Metabolomic and lipidomic analyses revealed that *cis*-TSG influenced key pathways, notably glycerophospholipid metabolism, sphingolipid metabolism, and bile acid biosynthesis. The treatment with *cis*-TSG increased monounsaturated and polyunsaturated fatty acids (MUFAs and PUFAs), enhancing peroxisome proliferator-activated receptor alpha (PPARα) activity. Transcriptomic data confirmed these findings, showing the downregulation of genes linked to lipid metabolism, inflammation, and oxidative stress in the *cis*-TSG-treated group. The findings suggest that *cis*-TSG has a hepatoprotective effect through modulation of lipid metabolism and PPARα activation.

## Graphical Abstract

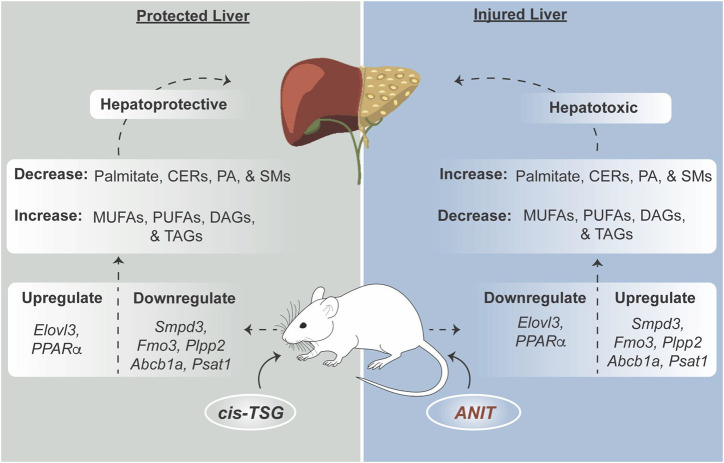

## 1 Introduction

*Polygonum multiflorum* (PM) is a traditional Chinese medicinal herb with a rich history of use for various health benefits, including hepatoprotection, lipid regulation, hair coloring and growth, bone loss attenuation, cardioprotection, neuroprotection, anti-cancer properties, anti-aging effects, and antioxidant properties (Lin et al., 2018; Shin et al., 2020; Teka et al., 2021; Ham et al., 2019). Recent studies have further elucidated the pharmacological effects of different PM extracts and isolated compounds, such as TSG (Yang et al., 2005; Zhao et al., 2005; Liang et al., 2010; Dong et al., 2014; Qian et al., 2020), emodin, rhein (Liu et al., 2009; Zhou et al., 2015; Dong et al., 2016; Sun et al., 2016), and polysaccharides (Zhu et al., 2016).

However, the hepatotoxicity of PM has drawn attention, with conflicting findings on the responsible compounds, including stilbene glycosides (Wang Q. et al., 2022; Wang S. et al., 2022), anthraquinones (Hu et al., 2021; Wang S. et al., 2022), and tannins (Teka et al., 2021). Despite concerns about its hepatotoxicity, PM has also been noted for its hepatoprotective effects (Lee et al., 2012), which highlights the plant’s dual nature in terms of liver health. This duality of hepatotoxicity and hepatoprotection warrants further in-depth research. In a recent untargeted NMR-based metabolomic study on PM’s hepatotoxicity, a non-linear hepatotoxic effect was identified, attributed to the hepatoprotective effects of some of its constituents. This finding suggests a balance between hepatotoxic and hepatoprotective effects (Ruan et al., 2019). In PM, *trans*-TSG is the primary stilbene compound, while its stereoisomer *cis*-TSG occurs naturally in low percentages, but its levels increase during the repetitive steaming and drying process of PM (Wenping et al., 2022).

Alpha-naphthyl isothiocyanate (ANIT) is a toxic compound commonly used to develop intrahepatic cholestasis (IC) models in mice, effectively mimicking human chronic cholangitic diseases (Dai et al., 2018). A single dose of ANIT disrupts the epithelial cells of the bile duct, leading to neutrophil infiltration, excessive necrosis, and bile duct obstruction (Cullen et al., 2016). The accumulation of bile acids results in mitochondrial dysfunction, oxidative stress, and lipid peroxidation damage, contributing to the development of cholestasis fibrosis (Khayat et al., 2023). Therefore, interventions that alleviate these interconnected pathophysiological responses can help reduce hepatocyte damage and confer hepatoprotection. Previous studies have shown that PM can prevent liver damage induced by cholestasis by modulating bile acid-related protein expressions and controlling redox states, thereby maintaining hepatic bile acid homeostasis (Teka et al., 2021). However, there is no research study reported on the responsible components for PM hepatoprotection activity using ANIT-induced animal models.

Metabolomics is an emerging discipline within the broader omics domain, offering significant potential for capturing detailed cellular dynamics (Wang R. et al., 2020). Typically performed by targeting specific cells, tissues, or entire organisms (McKay, 2023), metabolomics is dedicated to identifying small molecule metabolites to analyze pathological and physiological processes in of complex biological systems. This method often utilizes liquid chromatography-mass spectrometry (LC-MS) due to its powerful quantitative and qualitative determination capabilities and high sensitivity (Wang L. M. et al., 2020; Tan et al., 2021). Metabolomics integrates the competencies of analytical chemistry, biochemistry, and statistics to provide strategies for understanding dynamic quantitative changes in metabolite levels (McKay, 2023; Gao and Xiao, 2023). Advances in science and technology over the past decades have ushered us into the era of multi-omics. Despite being at an earlier developmental stage than genomics, transcriptomics, and proteomics, metabolomics offers the distinct advantage of studying cellular entities that most directly influence the end phenotype (McKay, 2023). Today, metabolomics is a powerful method for unveiling the mechanisms of bioactive compounds, aiding in their development as a drug (Zhang et al., 2019; Tan et al., 2021).

The primary objective of this research study was to examine the hepatoprotective effect of *cis*-TSG and elucidate potential underlying mechanisms against ANIT-induced cholestasis in mice. Both untargeted and targeted metabolomics approaches were employed (Wang R. et al., 2020), as their combination is crucial for uncovering and accurately identifying differential metabolites, enabling a comprehensive analysis of subsequent metabolic molecular markers (Lelli et al., 2021). Lipidomics, transcriptomics, real-time qPCR, and western blot-based research analyses. were then conducted to further investigate and confirm the metabolomics results.

## 2 Materials and methods

### 2.1 Materials

ANIT was obtained from Sigma-Aldarich Corporation (St. Louis, MO, United States). *Trans*-TSG, with a chemical purity of 99.8%, was bought from Shanghai Yuanye Bio-Technology Co., Ltd. (Shanghai, China). Alanine aminotransferase (ALT) and aspartate aminotransferase (AST) kits were procured from Nanjing Jiancheng Bioengineering Institute (Jiangsu, China). High-performance liquid chromatography (HPLC)-grade methanol, acetonitrile, and isopropanol were supplied by Thermo Fisher Scientific Co., Ltd. (Fair Lawn, NJ, United States), while mass spectrometry (MS)-grade formic acid was acquired from Anaqua Chemicals Supply (Wilmington, DE, United States). Diethyl pyrocarbonate (DEPC) water was provided by Applied Biosystems, Ambion, Life Technologies (Austin, TX, United States). TRIzol total RNA extraction and RNA purification kits were purchased from Shanghai Meiji Biotechnology Co. Ltd., and QIAzol Lysis Reagent was obtained from Qiagen (Germany). Biowest Agarose for agarose gel detection was procured from Biowest (Spain), and Illumina^®^ stranded mRNA and NovaSeq Reagent Kit were purchased from Illumina (United States). Metabolite and bile acid standards were purchased from Sigma-Aldrich Corporation (St. Louis, MO, United States), and ultrapure water was obtained from Watsons Food and Beverage Co., Ltd. (Guangzhou, China). All primers for reverse transcription quantitative PCR (RT-qPCR) and polyclonal antibodies for western blotting were provided by Affinity Biosciences (Cincinnati, OH, United States).

### 2.2 Preparation of *cis*-TSG

Due to the instability of *cis*-TSG in its solid state, it is not commercially available and must be isolated from PM Root extract or obtained by converting its isomer, *trans*-TSG, using sunlight. In this study, we used *trans*-TSG previously isolated and purified in our laboratory. The *trans*-TSG was extracted from PM (voucher number PMR-20160432) purchased from Tongrentang (Beijing, China) and authenticated by Professor Zhang Lijuan of Tianjin University of Traditional Chinese Medicine. It was isolated from the *n*-butanol extract of a 60% ethanol extract of PM using multiple chromatographic techniques, combined with NMR and MS identification methods, and its structural and molecular weight were verified against literature data. To prepare a 1 mg/mL solution of *trans*-TSG, 2 g of *trans*-TSG stored at −80°C was dissolved in 2L of double-deionized water (ddH_2_O) in a 4L transparent beaker. The solution was stirred continuously until fully dissolved and then exposed to sunlight for 1 h at room temperature. The production of *cis*-TSG was confirmed using an Agilent (DAD) HPLC system (Germany) with Waters COSMOSIL C_18_ packed column (4.6 mm × 250 mm, 5 μm). Isocratic elution with methanol (A, 35%) and water (B, 65%) as mobile phase at a flow rate of 1 mL/min was employed, with a total run time of 6–7 min. Upon achieving 55%–70% conversion, the solution was concentrated using a rotary evaporator. The product was further purified by preparative liquid chromatography at a flow rate of 60 mL/min using the same mobile phase. The sample volume was maintained at 400 mg/mL of ddH_2_O, and the detector wavelength was set at 320 nm to collect both *cis*-TSG and untransformed *trans*-TSG. Fractions were collected and dried immediately in a dark room to prevent light exposure, which destabilizes *cis*-TSG. Preparative liquid chromatography was conducted using a GILSON preparative liquid chromatography system (Gilson, Inc., United States), equipped with an NU3000 series UV detector (200–400 nm, dual-wavelength channel), an NP7000 series pump (0–100 mL/min, 10 Mpa), and an HPLCONE C_18_ column (50 mm × 250 mm, 10 μm) (Suyan, Science and Technologies, Bangalore, India). The purity of *cis*-TSG was confirmed using Agilent analytical UHPLC, resulting in a total yield of 2.5 g of *cis*-TSG with 98.5% purity, stored at −80°C for further experiments.

### 2.3 Drug preparation

The dosing rationale for stilbene glycosides is based on the maximum safe clinical dose of PM in adults, reported as 12 g/day (Lei et al., 2015). PM contains at least 1% stilbenes (approximately 2.6%) and about 0.2% emodin. According to the American human dose conversion guidelines, the clinical equivalent dose of TSG for mice is approximately 60 mg/kg, while that for emodin is 5 mg/kg (Lei et al., 2015). In this study, we administered a dose of 300 mg/kg, which is five times the clinical equivalent dose of TSG (Lei et al., 2015; Xu et al., 2017).

### 2.4 Animals

Male C57 BL6 mice (32 in total, weighing 20–25 g, SPF grade), aged 7–8 weeks, were purchased from Beijing Vital River Laboratory Animal Technology Co., Ltd. (Beijing, China). The mice were acclimatized for 1 week before the experiments. They were housed in an air-conditioned, specific pathogen-free facility under a controlled 12-h light/12-h dark cycle at 25 ± 2°C and 45 ± 5 humidity, with free access to chow and water. Body weights were recorded daily before drug administration. All animal studies were performed in compliance with the National Institutes of Health (NIH) and local guidelines at the Center for Laboratory Animals, Tianjin University of Traditional Chinese Medicine, Tianjin, China (Ethical number: TCM-LAEC202318m45631). The mice were randomly divided into four groups (n = 8). The treatment groups were as follows: (1) Group I: Control (CO) - administered only drug solvents. (2) Group II: *Cis*-TSG (CI) - administered only *cis*-TSG. (3) Group III: Model (MO) - administered ANIT on the 5th day. (4) Group IV: *Cis*-TSG + ANIT (CM) - administered *cis*-TSG for 7 days and ANIT on the 5th day. Drugs were dissolved in 0.5% sodium carboxymethyl cellulose (CMC-Na) solution and administered orally via gavage once daily for 7 days. Groups CI and CM received 300 mg/kg *cis*-TSG for seven consecutive days (Days 1–7), while groups CO and MO were administered 10 mL/kg CMC-Na via intragastric administration. On Day 5, 2 hours after the administration of CMC-Na or drugs, a single dose of ANIT (50 mg/kg, p. o. in corn oil) was given to MO and CM groups. The control group was given the vehicle orally. Forty-8 hours after ANIT treatment and 2 hours after the final drug administration on the 7th day, the mice were anesthetized with 65 mg/kg sodium pentobarbital and euthanized in an ultra-clean workbench to harvest all experimental tissues. Blood samples were collected by orbital bleeding into 1.5 mL heparinized tubes. Plasma was separated by centrifuging the blood at 13,200 × *g* for 10 min at 4°C. Following the collection of blood, the mice underwent dissection to obtain liver and distal ileum tissues. Subsequently, the liver tissue was washed, weighed, and divided into two parts for further analysis. One part was fixed in 4% paraformaldehyde at room temperature and stained with hematoxylin-eosin (H-E) for histopathological analysis. The other part was placed in pre-labeled cryogenic vials according to the mouse number in each group, snap-frozen in liquid nitrogen, and stored at −80°C until further analysis.

### 2.5 Biochemical and immunobiological analyses

Plasma levels of ALT and AST were measured using an automatic spectrophotometry biochemical analyzer (TECAN, Switzerland) with commercially available kits from Nanjing Jiancheng Bioengineering Institute, following the manufacturer’s protocol. Histological analysis was conducted to assess liver tissue morphology using H-E staining. Liver tissues were fixed in 4% paraformaldehyde, embedded in paraffin, and sectioned at a thickness of 6 μm. The sections were dehydrated using graded ethanol and xylene solutions. Nuclei were stained with 5% hematoxylin solution for 10 min, followed by rinsing with distilled water for 5 min. The samples were then incubated in 0.1% hydrochloric alcohol for 30 s and counter-stained with eosin solution for 2 min. After additional washing and dehydration, the H&E stained sections were mounted and imaged using a fluorescence microscope.

### 2.6 Metabolomics analysis

Blood samples from mice were centrifuged at 13,200 × *g* for 10 min at 4°C to obtain plasma. Pre-cooled acetonitrile (−20°C) was added to 100 μL of plasma, vortexed for 2–3 min, and centrifuged again for 15 min at 13,200 × *g* at 4°C to collect the supernatant. For liver tissue analysis, 100 mg of liver tissue was placed into a 2 mL grinding tube with 400 μL of pure water and 2-3 magnetic beads. The tubes were pre-cooled at −20°C for 20–30 min before homogenization at 60 Hz for 120 s. Next, 100 μL of the liver homogenate was transferred to a 1.5 mL centrifuge tube, and 400 μL of pre-cooled acetonitrile was added. The mixture was vortexed for 2–3 min, centrifuged at 13,200 × *g* for 15 min at 4°C, and the supernatant was collected. Then, in both plasma and liver sample preparation, the supernatant was dried under a liquid nitrogen flow and reconstituted with 100 μL of a 50% methanol-water mixture, vortexed, and centrifuged again at 13,200 × *g* for 20 min at 4°C. Approximately 70–80 μL of the supernatant was transferred to a new UHPLC vial for metabolomics analysis, and 2 μL of this supernatant was injected. A quality control (QC) sample, about 5–10 μL, was prepared using the same method and used for UHPLC-MS/MS metabolomic analysis. QC samples were injected at regular intervals throughout the analytical process to ensure repeatability (Lin et al., 2021). Data analysis included principal component analysis (PCA) and orthogonal partial least squares discriminant analysis (OPLS-DA) to analyze differences between groups. Differential metabolites and variable importance in projection (VIP) scores were determined, followed by KEGG, network, and pathway analyses based on the metabolite comparison data. Bile acids and selected lipids were further analyzed using targeted metabolomics and lipidomics, respectively.

### 2.7 Bile acid-targeted metabolomics analysis

In this experiment, 100 μL aliquots of plasma previously stored at −80°C were transferred into 1.5 mL Eppendorf tubes. The samples were allowed to thaw at 4°C before analysis. Each sample was supplemented with 2 μg/mL of cholic-2,2,4,4-d4 acid (CA-d4) as an internal standard (IS). Following thorough mixing, the samples were placed on ice for 5 min, and 1 mL of pre-cooled acetonitrile (maintained at −20°C) was added to each 1.5 mL Eppendorf tube. The resultant sample-acetonitrile mixture was then agitated for 30 min and subsequently centrifuged at 13,200 *g* and 4°C for 10 min. After centrifugation, the resulting supernatant was carefully transferred to a sterile centrifuge tube and desiccated under a stream of liquid nitrogen while eliminating the protein debris. The desiccated samples were then reconstituted by adding 50 μL of a 1:1 aqueous-methanol solution and vortexed for 3–5 min, following which they were centrifuged for 20 min at 13,200 × *g* and 4°C. Subsequently, 50–70 μL of the resulting supernatant from each centrifuge tube was carefully transferred to a UHPLC vial for targeted metabolomic analysis of bile acids using UHPLC-MS/MS. During the UHPLC-MS/MS analysis, an injection volume of 5 μL was employed. The same procedures were also followed to prepare the ileum tissues.

For liver samples, 50 mg of liver tissue was weighed and put into a 2 mL grinding tube. After adding 250 μL (five-fold of the weight) of pre-cooled water, 2-3 magnetic beads were inserted into each grinding tube. The tubes were then put into the holes of a previously cooled (at −20°C for 20–30 min) homogenizer’s grinding tube holder. The holder is placed in its proper position and tightly tied. The liver tissues were homogenized at 60 Hz for 2 min, and 250 μL of the homogenate liquid was then transferred into a 1.5 mL centrifugal tube. In each Eppendorf tube, 10 μL of the internal standard (2 μg/μL IS, CA-d4) was added, and the mixture was thoroughly vortexed and placed on ice for 5 min. To each tube, 1.25 mL of ice-cooled acetonitrile containing 3% ammonia was added, and the mixture was vortexed for 1 h to ensure thorough mixing. The mixture was then centrifuged for 10 min at 4°C at 13,200 × *g*. The supernatant was transferred to a new Eppendorf tube, and the precipitate was discarded. After drying the supernatant on a liquid nitrogen flow, 50 μL of aqueous-methanol solution (1:1) was used to re-constitute the samples. Samples were then vortexed (well mixed) and centrifuged for 20 min at 4°C, 13,200 × *g*. The supernatant volume of 50–70 μL was transferred into a UHPLC-MS/MS vial, and a 5 μL sample of each was used for BA determination during the UHPLC-MS/MS run.

### 2.8 Lipidomic analysis

Plasma samples stored at −80°C were thawed at 4°C. A 100 μL aliquot of each plasma sample was transferred into a new Eppendorf tube, and 300 μL of pre-cooled isopropanol was added. The mixture was vortexed for 5 min to ensure even mixing, then incubated at −20°C for 1 h. Following incubation, the samples were centrifuged at 13,200 × *g* for 20 min at 4 °C, and the supernatant was collected for UPLC-MS analysis. The QC sample was prepared by combining 10 μL of each plasma sample, gently shaking, and then adding isopropanol in a volume three times that of the total plasma sample. To analyze liver tissue, a 50 mg sample of liver tissue was weighed and homogenized with five volumes of pure water and 2-3 magnetic beads using a homogenizer at 60 Hz for 2 min. Following this, a 100 μL aliquot of the resulting homogenate was transferred to a new tube, after which 300 μL of pre-cooled isopropyl alcohol was added. The mixture was vortexed for 5 min, incubated at −20°C for 1 h, and then centrifuged at 13,200 × *g* for 20 min at 4°C. The supernatant was subsequently transferred to an AB vial for UHPLC-MS/MS lipidomic analysis.

### 2.9 Transcriptome and protein expression analysis

Mouse liver tissues were harvested and stored at −80°C. RNA was extracted from the liver tissues, and the quality of the extracted RNA was evaluated using the Agilent 2100 Bioanalyzer (Agilent Technologies, California, United States). Following this assessment, a cDNA library was constructed for transcriptome sequencing, and the analysis was performed as previously described (Kim et al., 2018). Data analysis included the following steps: differential gene expression was visualized using Venn diagrams and heatmaps. KEGG pathway analysis and Gene Ontology (GO) term analyses were conducted using Majorbio (https://www.majorbio.com) to identify enriched pathways and biological processes. Total mRNA was extracted from liver tissue using Trizol reagent, following the manufacturer’s guidelines. cDNA synthesis was later performed using primers and the Novoptotein reverse transcription kit, adhering to the provided protocol. Primers were designed and synthesized by Sangon Biotech. GAPDH was used as the internal control gene for normalization, and the relative mRNA expression of PPARα was assessed. For protein expression analysis of PPARα, 30 mg of liver tissue was weighed and processed following standard procedures. Each sample was homogenized in 300 μL of lysis buffer in a 2 mL grinding tube, crushed on ice, and then centrifuged for 10 min at 13,200 × *g* at 4°C. Protein concentration was determined using the bicinchoninic acid (BCA) method. Proteins were denatured by adding 4X loading buffer proportionally and heating at 100°C for 10 min using a digital double-heat biological dry bath (Thermo Scientific, Fisher, United States). The denatured proteins were stored at −80°C. Proteins were later separated by SDS-PAGE (10% sodium dodecyl sulfate-polyacrylamide gel electrophoresis) and transferred to a polyvinylidene fluoride (PVDF) membrane. The PVDF membrane was blocked and then probed with primary antibodies targeting PPARα (1:1,000; proteintech, PPARA-Antibody-66826-1-lg) and GAPDH (1:5,000). After incubation with primary antibodies, the membrane was treated with HRP-conjugated secondary antibodies. Detection was performed using a chemiluminescence (ECL) kit (EVERBRIGHT, Suzhou, China), and intensity analysis was conducted using ImageJ software (NIH, Bethesda, United States).

### 2.10 Statistical analysis

Statistical analyses were performed using GraphPad Prism (GraphPad Software, San Diego, CA, United States). Data are presented as means ± SD and visualized using bar charts. Statistical significance between the two groups was determined using Student’s t-test. For comparisons among multiple groups, One-way ANOVA was employed. A *p*-value of less than 0.05 was considered statistically significant.

## 3 Results

### 3.1 *cis*-TSG protects mice against ANIT-induced cholestasis

**FIGURE 1:**
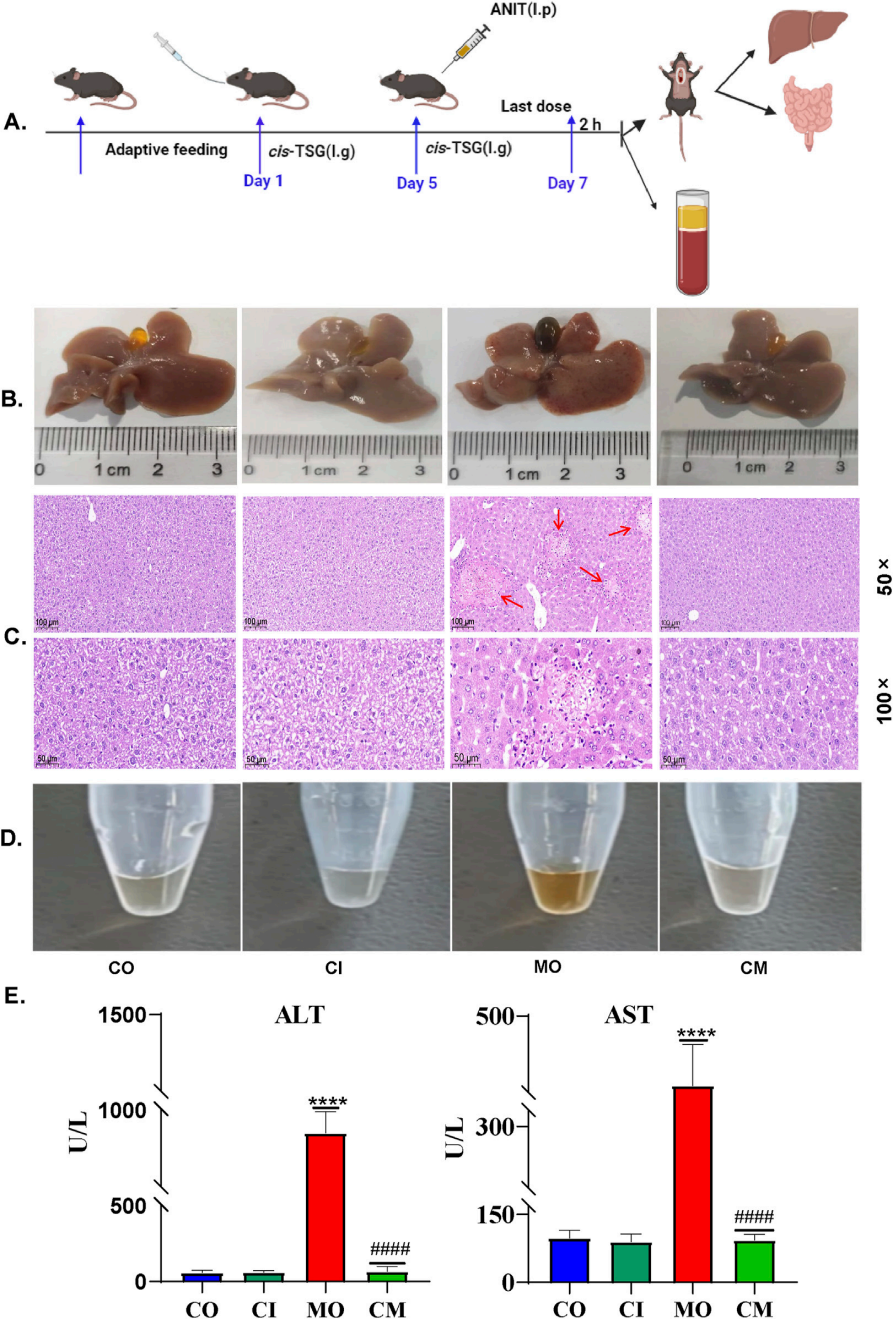
*cis*-TSG protected mouse against ANIT-induced cholestasis: **(A)** Experimental work flow in cholestasis animal model (n = 8 ). **(B)** Image of liver and gallbladder morphology showing the color and inflammation difference between the groups. **(C)** and **(D)** Representative HE staining pictures of the liver tissue with 50 × and 100 × magnifications respectively (n = 3). **(E)** Image of plasma fluid showing color difference between the groups. **(F, G)** Plasma levels of ALT (U/L) and AST (U/L). The data were expressed as mean ± SD, and were analyzed by one-way ANOVA. **** *P* < 0.0001 vs the control group and #### *P* < 0.0001 vs the model group, n = 8. CO-control group, MO-model (ANIT) group, CI-(only cis-TSG) group, and CM-(ANIT + cis-TSG) group.

To investigate whether *cis*-TSG, a type of stilbene compound in PM, could worsen liver injuries in patients with a history of liver problems who take PM for its therapeutic benefits, we induced a cholestasis model in mice using ANIT. The enzymes ALT and AST are key indicators of liver diseases. To assess whether *cis*-TSG protects the liver during cell damage and cholestasis, we measured levels of ALT and AST in the plasma. The results showed that, compared to the model group, plasma levels of ALT and AST (U/L) were significantly lower in the *cis*-TSG treatment (Figure 1E, *p* ≤ 0.0001), almost similar to their level in the control group. Furthermore, the group administered with *cis*-TSG only exhibited comparable levels of ALT and AST as the control group. This suggests that *cis*-TSG does not induce any discernible toxic effects on its own. Upon comparing the CI group with both the CM and MO groups, it becomes evident that the significant reduction of ALT and AST levels in the CM group is attributed solely to the administration of *cis*-TSG. In Figure 1B, the ANIT-induced group displayed distinct pathomorphological features characterized by visible inflammation indicative of cholestasis. However, treatment with *cis*-TSG in ANIT-induced mice markedly improved the pathological features, demonstrating similarity to the control group (Figure 1B). Further histological analysis with H&E staining revealed that *cis*-TSG treatment effectively prevented inflammatory cell infiltration and hepatocyte damage induced by ANIT (Figure 1C). In addition to our findings, we observed changes in bile color (Figure 1B), a characteristic feature of liver cholestasis, and plasma color (Figure 1D), which can indicate the effect of *cis*-TSG. In comparison to the control group, ANIT treatment resulted in significantly darker bile color, likely attributed to disruptions in bile acid homeostasis caused by ANIT treatment. The perturbed composition of bile acids was restored to near-physiological levels following *cis*-TSG treatment, as evidenced by the observed change in bile color in the *cis*-TSG treatment groups as compared to the control and model groups (Figure 1B). These data demonstrate that *cis*-TSG effectively reduced plasma ALT and AST levels, ameliorated liver injury, and impeded inflammatory responses by inhibiting inflammatory cell infiltration. This suggests that *cis*-TSG exerts a hepatoprotective effect in ANIT-induced cholestasis.

### 3.2 *cis*-TSG treatment modulates unique metabolic profiles

**FIGURE 2:**
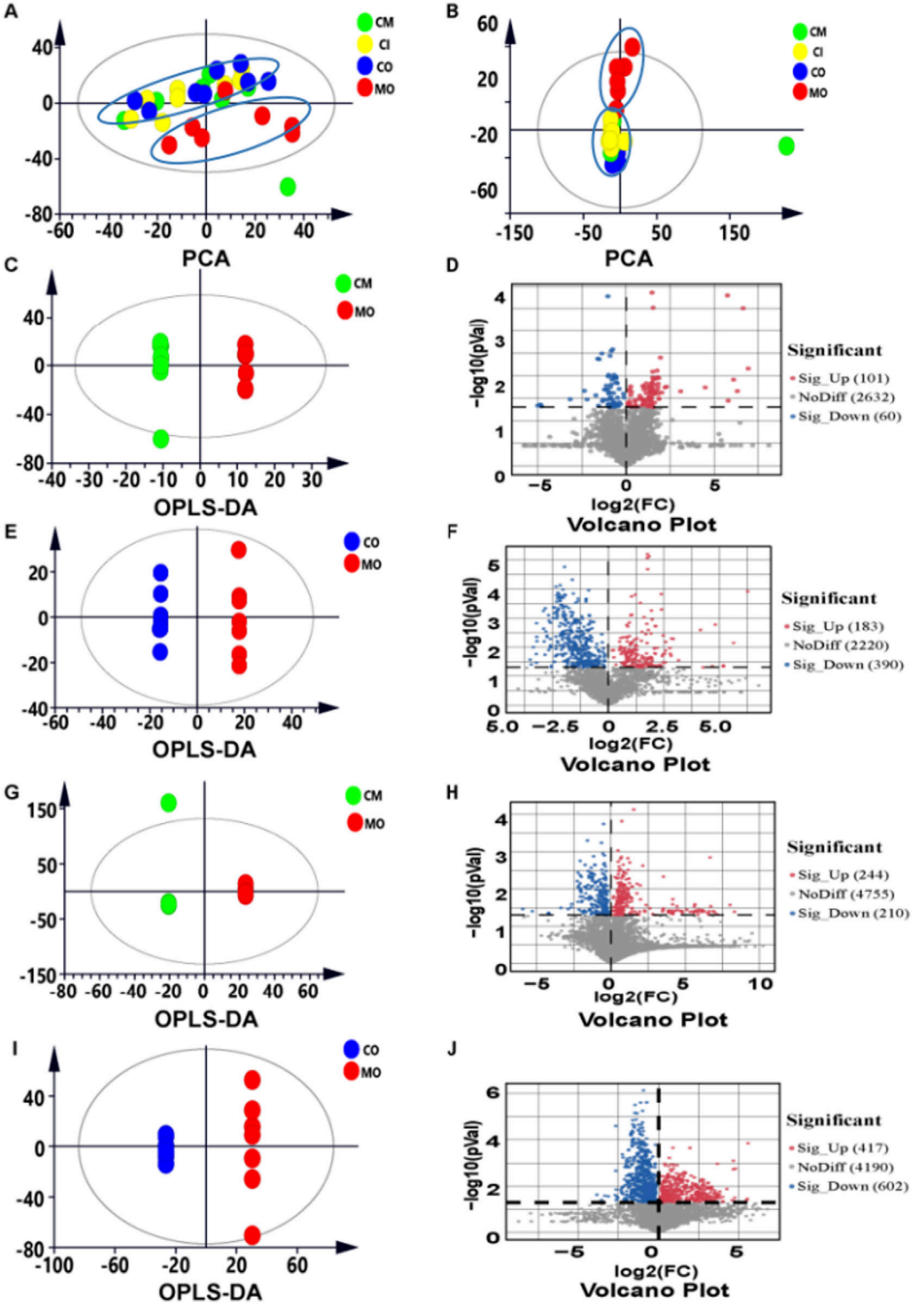
*cis*-TSG r estor ed significant number of metabolites alter ed by ANIT: **(A)** PCA score plot of liver metabolite profiling between the four groups. **(B)** PCA score plot of plasma metabolite profiling between the four groups. **(C)** OPLS-DA score plot of liver metabolite profiling between the MO and CM groups **(D)** Volcano plot showing significantly changed metabolites between the two groups. **(E)** OPLS-DA score plot of liver metabolite profiling between the CO and MO groups. **(F)** Volcano plot showing significantly changed metabolites between the two groups. **(G)** OPLS-DA score plot of plasma metabolite profiling between the MO and CM groups. **(H)** Volcano plot showing significantly changed metabolites between the two groups. **(I)** OPLS-DA score plot of plasma metabolite profiling between the CO and MO groups. **(J)** Volcano plot showing significantly changed metabolites between the two groups.

To investigate the metabolic profiles associated with *cis*-TSG treatment, data collected from UHPLC-MS in both positive and negative ion modes were further processed using Compound Discoverer 3.0 software and Metaboanalyst 6.0 (https://www.metaboanalyst.ca/). Multivariate statistical analysis was conducted, and the PCA plots for liver (Figure 2A) and plasma (Figure 2B) samples were generated. PCA score plots demonstrated distinct clustering of QC samples, indicating high instrument reproducibility and stability. Notable differentiation was observed between the CO and MO groups, while no clear separation was seen among the CO, CI, and CM groups. This suggests that *cis*-TSG confers hepatoprotection by altering the metabolite profile to resemble that of the control group, both in the liver and plasma. OPLS-DA score and volcano plots further confirmed the distinct separation between the control and model groups in the liver (Figures 2E, F) and plasma (Figures 2I, J). Similarly, clear differentiation was observed between the model and *cis*-TSG pretreated group in the liver (Figures 2C, D) and plasma (Figures 2G, H). These results show significant changes in the metabolic profile due to *cis*-TSG treatment. Potential metabolic biomarkers related to the effects of *cis*-TSG were screened using pairwise comparisons of OPLS-DA. Significant metabolites were identified based on a VIP threshold (VIP >1.0) and *p*-value (*p* < 0.05), comparing CO and MO and MO and CM groups. These significant metabolites were further identified by comparison with standards, internal databases (mzCloud, Chemical Spider, etc.), and online mass spectral databases (HMBD, METLIN, Pubchem) and validated via KEGG identification. The key hepatoprotective metabolites are summarized in Table 1. In the model group, LPCs and BAs were elevated in the liver and plasma, while these metabolites were reduced by *cis*-TSG treatment. Conversely, the levels of 5-OxoETE, 12,13-DHOME, and 9,10-DHOME were decreased in the MO group but increased in the CM group, showing enhanced fatty acid oxidation effect with *cis*-TSG treatment. Seven metabolites, including LPC (18:2), LPC (18:3), LPC (20:5), and taurocholic acid, were significantly higher in the plasma of the MO group compared to the CM group, with *cis*-TSG pre-treatment reversing their increase. Collectively, these results highlight the differential influence of *cis*-TSG on potential metabolic pathways, underscoring its hepatoprotective effects in ANIT-induced cholestasis.

**TABLE 1:** Hepatoprotection-related candidate biomarkers and their fold changes.

S.No.	Metabolites	RT	m/z	MO vs*.* CO	CM vs*.* MO	Detected	HMDB	Biological matrices
1	Choline	0.83	103.1000	↓	↑	LC-MS	HMDB0000097	liver
2	2-(S-Glutathionyl)acetyl-glutathione	2.10	654.1626	↑	↑	LC-MS	HMDB0060343	plasma
3	Hexanoylcarnitine	3.49	259.1783	↓	↓	LC-MS	HMDB0000756	plasma
5	Taurocholic acid	4.90	515.2917	↑	↓	LC-MS	HMDB0000036	Plasma/liver
6	α-Muricholic Acid	5.26	444.2645	↑	↓	LC-MS	HMDB0000506	liver
7	β-Muricholic Acid	5.26	444.2645	↑		LC-MS	HMDB0000865	liver
8	Sphingosine	5.72	299.2820	↑	↓	LC-MS	HMDB0000252	plasma
9	Tauroursodeoxycholic acid	5.78	499.2973	-	↓	LC-MS	HMDB0000874	liver
10	Taurochenodesoxycholic acid	5.79	516.3232	-	↑	LC-MS	HMDB0000951	liver
11	Sphingosine 1-phosphate	6.14	379.2486	↑	↓	LC-MS	HMDB0000277	plasma
12	LysoPC(20:5/0:0)	6.32	541.3160	↑	↓	LC-MS	HMDB0010397	plasma
13	LysoPC(18:3)	6.38	517.3162	↑	↓	LC-MS	HMDB0010388	plasma
14	LysoPC(0:0/20:4)	6.74	543.3311	-	↓	LC-MS	HMDB0061699	plasma
15	LysoPC(0:0/18:2)	6.75	519.3316	↑	↓	LC-MS	HMDB0010386	plasma
16	3-Oxotetradecanoic acid	6.97	242.1879	↓	↑	LC-MS	HMDB0010730	Plasma
17	3-Hydroxytetradecanoic acid	7.33	244.2036	-	↑	LC-MS	HMDB0061656	plasma
18	3-Oxohexadecanoic acid	7.66	270.2196	↓	↑	LC-MS	HMDB0010733	plasma
19	Oxybutynin	8.50	357.2299	↓	↑	LC-MS	HMDB0015195	plasma
20	5-OxoETE	8.64	318.2195	↓	↑	LC-MS	HMDB0246835	plasma
21	4,7,10,13,16-Docosapentaenoic acid	8.77	330.2561	↓	↓	LC-MS	HMDB0246621	plasma
22	9-Oxooctadecanoic acid	8.80	298.2508	↓	↑	LC-MS	HMDB0030979	plasma
23	N-arachidonyl glycine	8.65	361.5182	↓	↑	LC-MS	HMDB0005096	plasma
24	9,10-DHOME	8.81	296.2346	↓	↑	LC-MS	HMDB0004704	plasma
25	Pantothenic acid	2.44	219.11051	↑	↓	LC-MS	HMDB0000210	plasma
26	12,13-DHOME	9.41	296.2351	↓	↑	LC-MS	HMDB0004705	plasma

“↑” Activation of model vs. control or *cis*-TSG, vs*.* model; “↓” Inhibition of model vs. control or *cis*-TSG, vs. model (*p* < 0.05, and VIP >1). * identified by comparing with standard. – is not significant.

### 3.3 *cis*-TSG potentially modulates lipid metabolism and bile acid biosynthesis pathways to relieve cholestasis

**FIGURE 3:**
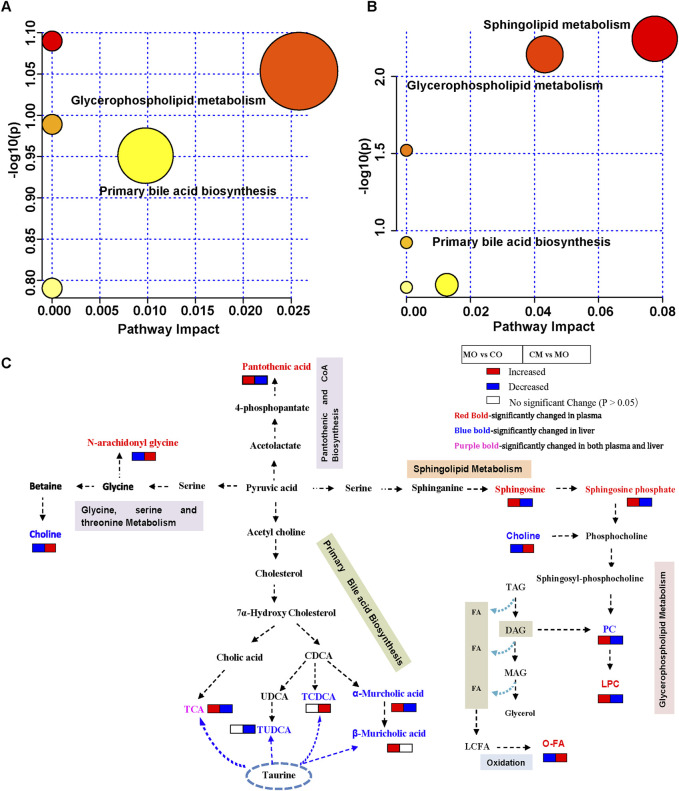
*cis*-TSG induced liver protection by modulating lipid and bile acid metabolism pathways. **(A)** Pathway analysis of identified liver differential metabolites between the MO and CM groups. **(B)** Pathway analysis of identified liver differential metabolites between the MO and CM groups. **(C)** Potential metabolic pathway for the hepatoprotection of cis-TSG based on significantly altered metabolites between the two groups. TCA, Taurocholic acid; CDCA, Chenodeoxycholic acid; UDCA, Ursodeoxycholic acid; TCDCA, Taurochenodeoxycholic acid; TAG, Triacylglycerol; DAG, Diacylglycerol;MAG, Monoacylglycerol; FA, Fatty acid; LCFA, Long chain fatty acid; O-FA, beta-oxigenated fatty acid..

To understand the metabolic pathways influenced by *cis*-TSG in alleviating cholestasis, we conducted a detailed metabolic pathway analysis comparing the model group with the *cis*-TSG-treated group. The analysis revealed significant associations with glycerophospholipid metabolism, sphingolipid metabolism, and primary bile acid biosynthesis pathways (Figures 3A, B). The sphingolipid metabolism pathway involves key metabolites such as sphingosine and sphingosine-1-phosphate. In the glycerophospholipid metabolism pathway, five metabolites, including choline and four lysophosphatidylcholines (LPCs), were identified. A notable number of fatty acids were involved in these pathways, highlighting the role of lipid and fatty acid metabolism in the hepatoprotective effects of *cis*-TSG (Table 1). Further investigation into these metabolic processes suggested that the modulation of lipid and fatty acid metabolism is central to the protective effects observed with *cis*-TSG treatment. Based on the plasma and liver metabolic profiling, a potential pathway of metabolic changes was outlined (Figure 3C). These findings underscore the importance of lipid (glycerophospholipid and sphingolipid) metabolism and bile acid biosynthesis in the therapeutic action of *cis*-TSG against cholestasis. By altering these metabolic pathways, *cis*-TSG appears to restore metabolic balance, thereby reducing liver damage and inflammation. Collectively, these results provide insight into how *cis*-TSG modulates critical metabolic pathways to exert its hepatoprotective effects in ANIT-induced cholestasis. The involvement of glycerophospholipid, sphingolipid, and bile acid biosynthesis pathways suggests that *cis*-TSG targets lipid and bile acid metabolism, reinforcing its potential as a therapeutic agent for liver diseases.

### 3.4 *cis*-TSG reduces bile acid accumulation in ANIT-induced cholestatic mouse

**FIGURE 4:**
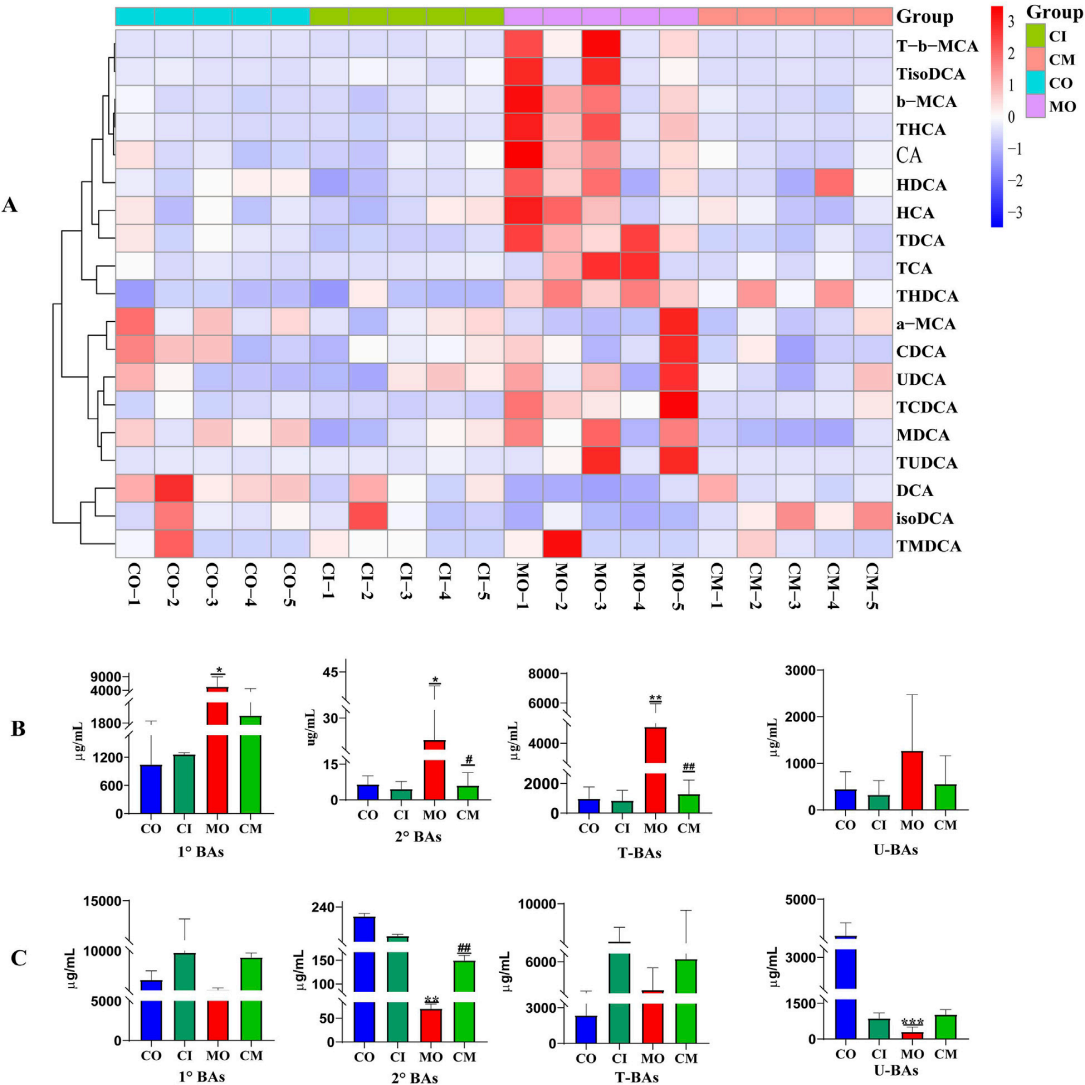
*cis*-TSG pr events ANIT-induced liver injur y by r egulating bile acid metabolism. **(A)** Correlation heatmap showing associations between differential liver concentration of 19 identified bile acids. **(B)** Liver concentration of bile acids between the four groups. **(C)** Distal ileum concentration of bile acids between the four groups. T-b-MCA, Tauro-beta-muricholic acid; TisoDCA, Tauroisodeoxycholic acid; b-MCA, beta-muricholic acid; THCA, Taurohyocholic acid/Tauro-γ-muricholic Acid; CA, Cholic acid; HDCA, Hyodeoxycholic acid; TDCA, Taurodeoxycholic acid; TCA, Taurodeoxycholic; TCA, Taurocholic acid; THDCA, Taurohyodeoxycholic acid; a-MCA, alpha-muricholic acid; CDCA, Chenodeoxycholic acid; UDCA, Ursodeoxycholic acid; TCDCA, Taurochenodeoxycholic acid; MDCA, Murideoxycholic acid; TUDCA, Tauroursodeoxycholic acid; DCA, Deoxycholic acid; isoDCA/3-DCA, isodeoxycholic acid (3-deoxycholic acid); TMDCA, and Tauromurideoxycholic acid. CO, control group; CI, cis-TSG only group; CM, cis-TSG + ANIT group; MO, ANIT group.

In a cholestatic mice model, it was found that ANIT induces a blockage of bile elimination from liver cells to the bile duct, leading to an increase in bile concentration within the liver (Zhang K. et al., 2021). To investigate the impact of *cis*-TSG on bile acid accumulation in the liver, an analysis of bile acid levels was conducted using a heatmap and *t*-test analysis in GraphPad. The heatmap (Figure 4A) as well as the bargraphs (Figure 4B) revealed that the model group exhibited the highest concentration of bile acids when compared to the CO, CI, and CM groups, indicating cholestatic liver toxicity within the model group. Additionally, the administration of *cis*-TSG significantly reduced the accumulation of bile acids in the liver, confirming its hepatoprotective effect against cholestatic liver injury. Furthermore, an analysis of several primary and secondary BAs revealed increased levels in the model group (Supplementary Figure S1). In comparison to the CO group, the levels of 6 primary BAs, including cholic acid (CA), HCA, taurocholic acid (TCA), beta-muricholic acid (*β*-MCA), tauro-(*α/β)-*muricholic acid (TMCA), and taurohyocholic acid/tauro-γ-muricholic acid (THCA), significantly increased, while the levels of alpha-muricholic acid (*α*-MCA) and chenodeoxycholic acid (CDCA) were notably decreased in the livers of cholestatic mice. Following treatment with *cis*-TSG, the levels of all 6 primary BAs reversed, displaying a significant downward trend. In the distal ileum tissue (Figure 4C)., concentration of bile acids were low in the model group which indicates their blockage and accumulation in the liver. However, *cis*-TSG restored normal flow of the bile acids and their concentration were comparable to the normal control in the ileum tissue. These results demonstrate that *cis*-TSG regulates and alleviates ANIT-induced liver injury by significantly reducing the concentration of bile acids in the liver.

### 3.5 *cis*-TSG reduces lipotoxicity in ANIT-induced cholestatic mouse

**FIGURE 5:**
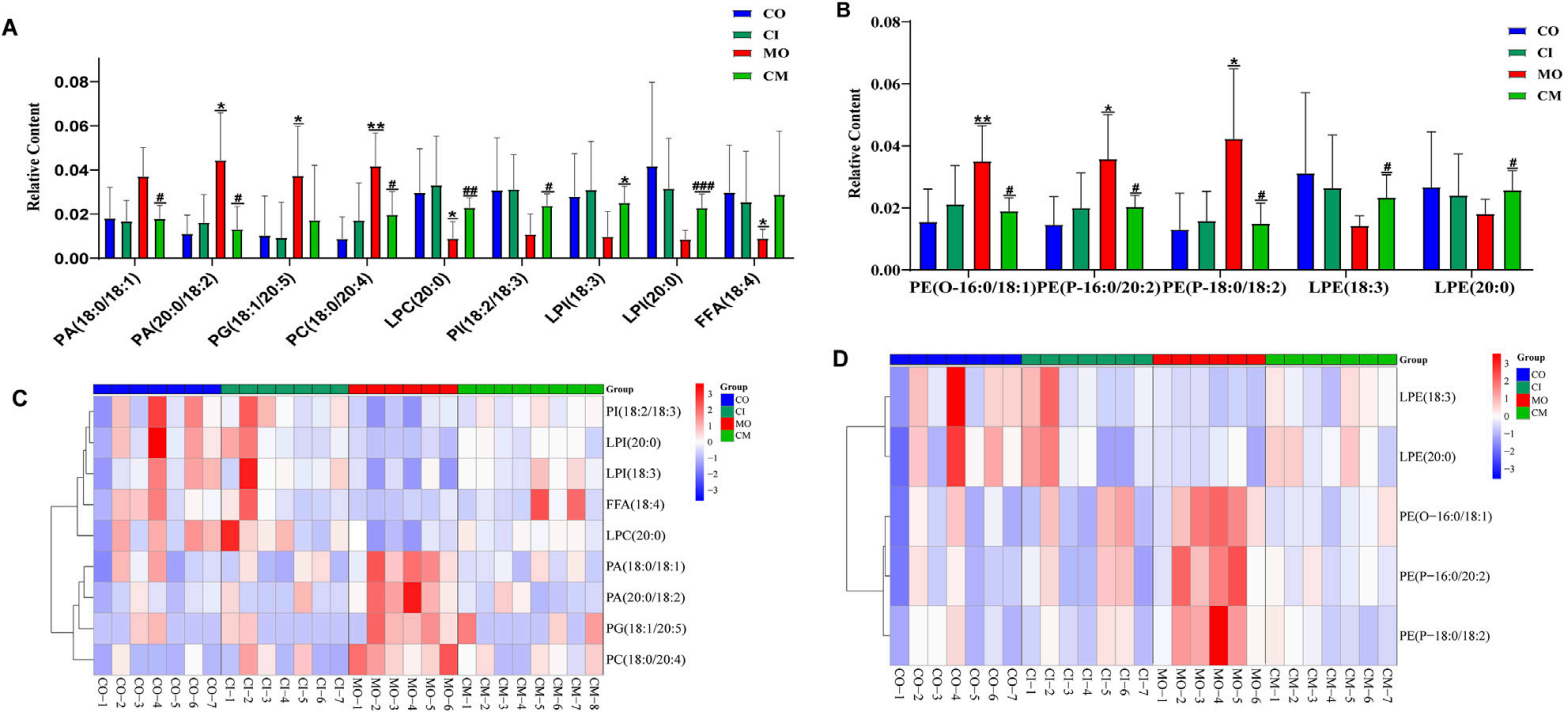
*cis*-TSG treatment alleviated liver injury by modulating accumulation of lipid metabolites in cholestasis mouse liver liver: **(A)** Concentration varaition of PA, phosphatidic acid; PC, Phosphatidylcholine; LPC, Lysophosphatidylcholine; PG, Phosphatidyl glycerol; PI, phosphatidylinositol; and LPI, Lysophosphatidyl inositol between the four groups. **(B)** Heatmap of the differential lipids in graph A. **(C)** Concentration variation of PE, Phosphatidyl ethanolamine and LPE, Lysophosphatidyl ethanolamine. **(D)** Heatmap of the lipids in graph C. CO, control group; CI, *cis*-TSG only group; CM, *cis*-TSG + ANIT group; MO, ANIT group.

**FIGURE 6:**
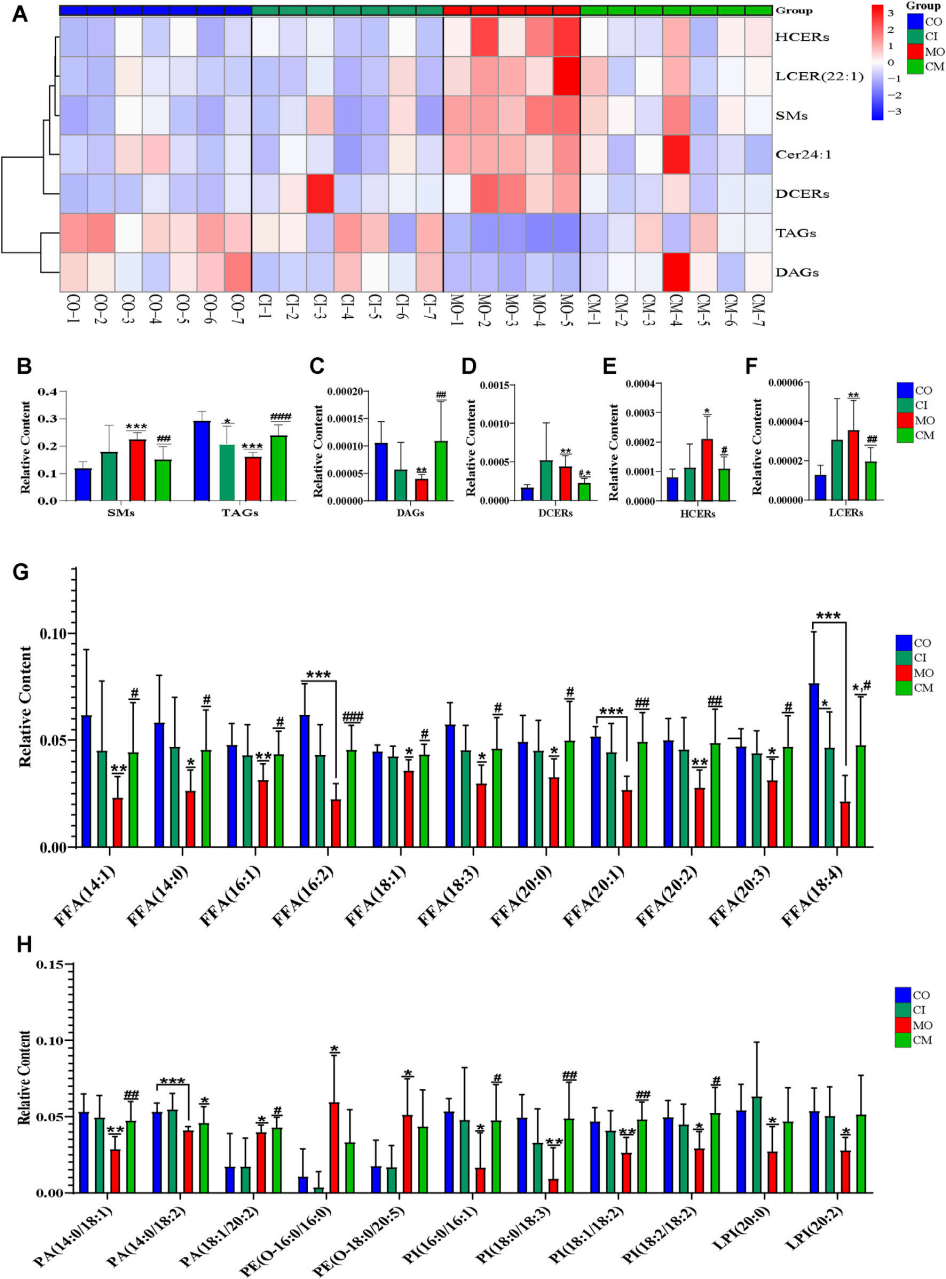
*cis*-TSG mitigated lipotoxicity via reducing ceramides and increasing MUFAs as well as PUFAs in cholestasis mouse. **(A)** Correlation heatmap showing associations of DCERs, Dihydroceramides; HCERs, Hexosylceramides; and LCER (22:1),Lactosylceramide, Cer 24:1, N-15Z-tetracosenoyl-sphing-4-enine; TAGs, Triacylglycerols; and DAGs, Diacylglycerols between the four groups in liver. **(B)** Relative liver concentration of TAGs, DAGs, DCERs, HCERs, and LCER (22:1) between the four groups. **(C)** Relative plasma concentration of FFA14:0, Myristic acid; FFA14:1, Myristoleic acid; FFA16:1, Palmitoleic acid; FFA16:2, Hexadecadienoic acid; FFA18:1, Oleic acid; FFA18:3, α-linolenic acid (ω-3); FFA18:4, Stearidonic acid (ω-3); FFA 20:0, Arachidic acid; FFA20:1, Eicosenoic acid(ω-9); FFA20:2, Eicosadienoic acid (ω-6); FFA20:3-Mead acid (ω-9) between CO, CI, MO, and CM and TM groups in plasma samples. **(D)** Relative plasma concentration of PA, phosphatidic acid; PE, Phosphatidyl ethanolamine; PI, phosphatidylinositol; and LPI, Lysophosphatidyl inositol between the four groups. CO, control group; CI, cis-TSG only group; CM, *cis*-TSG + ANIT group; MO, ANIT group.

Cholestatic liver disease disrupts the absorption and metabolism of lipids (Fu et al., 2019). To understand the impact of *cis*-TSG on lipid metabolism in cholestatic mice, we analyzed the concentrations of differential lipids using heatmap visualization and *t*-test analysis in GraphPad. Significant variations in lipid concentrations were observed between the control and model groups. Pre-treatment with *cis*-TSG reversed these changes, whereas the model group showed elevated levels of various lipids, indicating lipotoxicity. In liver tissues, the concentration of phosphatidic acid (PA) [PA (20:0/18:2) and PA (18:0/18:1)], a crucial precursor of most phospholipids, was significantly higher in the model group compared to the control group (Figure 5A). Treatment with *cis*-TSG completely reversed this elevation, restoring PA levels to those seen in the control group. Additionally, the concentrations of phosphatidylcholine (PC) and phosphatidylglycerol (PG) were significantly increased in the model group. *Cis-TSG* treatment effectively reversed these changes, normalizing PC and PG levels. The heatmap demonstrated a similar pattern, showing distinct differences in lipid concentrations between the study groups (Figure 5B). Phosphatidylethanolamines (PEs) such as P-18:0/18:1, O-16:0/18:1 and P-16:0/20:2 showed higher concentrations in the model compared to the control and *cis*-TSG-treated groups (Figures 5C, D). However, *cis*-TSG increased the concentrations of phosphatidylinositol (PI) and lysophosphatidylinositol (LPI) to levels similar to the control group (Figure 5A), indicating its role in restoring normal lipid metabolism. The levels of ceramides (CERs) and other damaging sphingolipids, such as dihydroceramides (DCERs), hexosylceramides (HCERs), and lactosylceramide (LCER (22:1), were found to be significantly elevated in the model group, consistent with previous studies (Choi et al., 2021). Furthermore, the total concentration of other sphingolipids, such as Sphingomyelins (SMs), also showed an increase in the model group (Figure 6A), indicating a dysregulation of sphingolipid metabolism in cholestatic liver injury. However, treatment with *cis*-TSG led to a reduction in the concentration of sphingolipids, thereby mitigating cholestasis-induced lipotoxicity and promoting liver recovery, as evidenced by improved liver health parameters. On the other hand, the amounts of triacylglycerols (TAGs) and diacylglycerols (DAGs) were significantly lower in the model group compared to the control, while these lipids were notably increased in the group treated with cis-TSG, bringing them more in line with the levels observed in the control group (Figure 6B). This may indicate a way to reduce the high concentrations of CERs by diverting them to the biosynthesis of FFAs and then safely storing them as DAGs and TAGs (Choi et al., 2021). These findings suggest that cis-TSG has a protective effect by adjusting lipid levels and reducing lipotoxicity in ANIT-induced cholestasis. These results underscore the potential therapeutic role of cis-TSG in improving cholestatic liver injury through the regulation of sphingolipid metabolism.

### 3.6 *cis*-TSG restored the production of MUFAs and PUFAs to mitigate lipotoxicity in ANIT-induced cholestatic mice

To evaluate the hepatoprotective effects of *cis*-TSG, we analyzed the concentrations of several monounsaturated fatty acids (MUFA) and polyunsaturated fatty acids (PUFA) in ANIT-induced cholestatic mice. As shown in Figure 6, the levels of these unsaturated fatty acids were significantly lowered in the model group compared to the control group. However, *cis*-TSG treatment significantly increased the concentrations of many MUFAs and PUFAs, confirming its protective effect against ANIT-induced cholestasis in mice. Additionally, the concentrations of various phosphatidylinositol (PI) and lysophosphatidylinositol (LPI) lipids were significantly reduced in the model group compared to the control. In the *cis*-TSG-treated group (CM), these lipid concentrations were almost completely restored to control levels, further indicating the hepatoprotective properties of *cis*-TSG. These findings collectively demonstrate that *cis*-TSG exerts a hepatoprotective effect by modulating the concentrations of various lipids, restoring normal lipid metabolism, and reducing lipotoxicity in ANIT-induced cholestasis.

### 3.7 cis-TSG protects the liver via downregulating genes related to lipotoxicity

**FIGURE 7:**
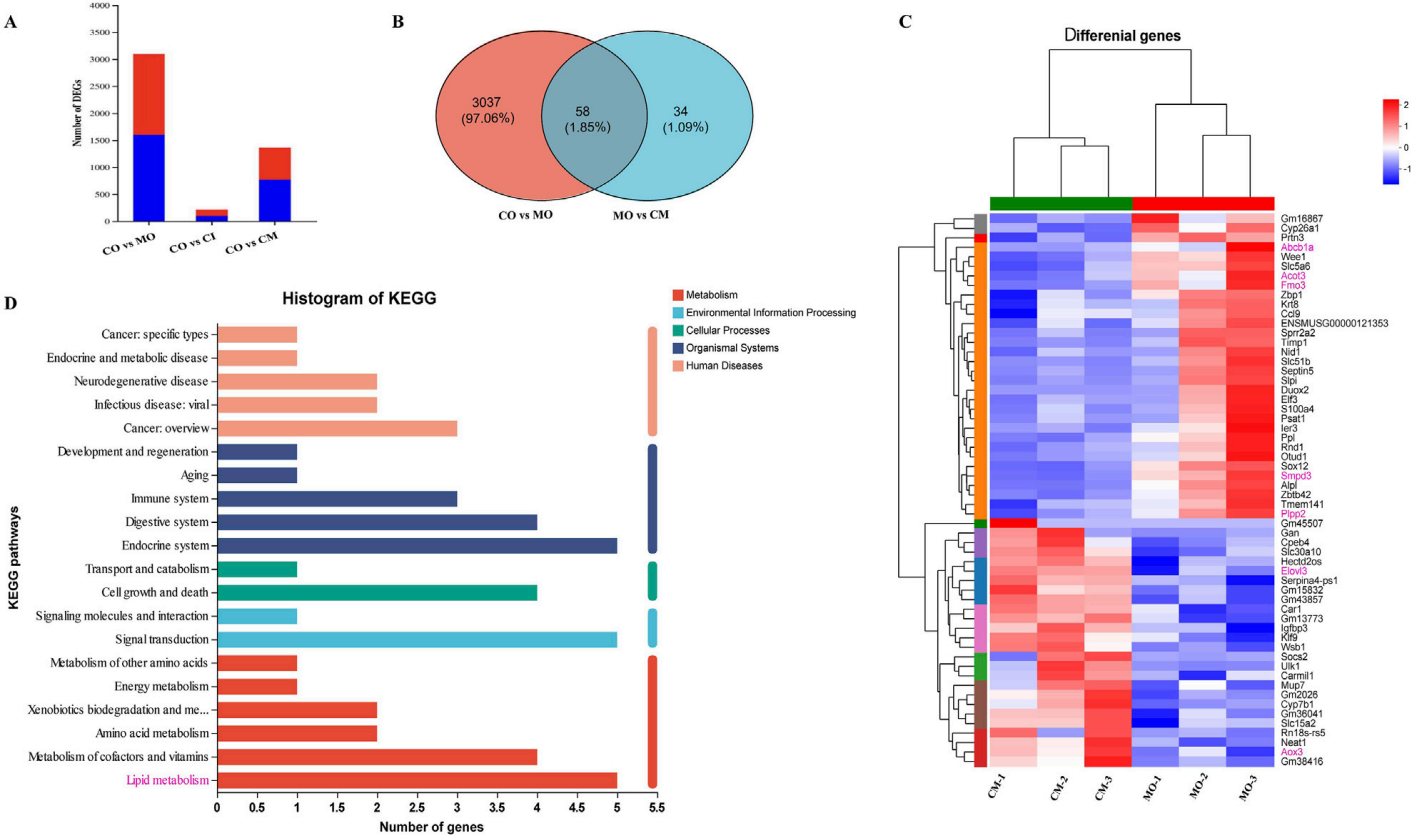
*cis*-TSG protected liver injury via regulating expression of genes related to lipid homeostasis in ANIT-induced cholestasis mouse. **(A)** Bargraph showing Number of genes altered in the MO, CI, and CM groups as compared to the normal control. **(B)** Venn diagram showing genes altered in common between CO vs MO and MO vs CM. **(C)** Correlation heatmap showing differentially altered genes between the CM and MO groups. **(D)** Histogram of metabolic pathway KEGG enrichment analysis showing correlation of critically influenced pathways with number significantly altered genes. CI, cis-TSG only group; CM, *cis*-TSG + ANIT group; MO, ANITgroup.

To elucidate the protective mechanisms of *cis*-TSG in ANIT-induced cholestasis, transcriptomic analysis was performed. In the model group, 3,095 genes were significantly altered, with 1,492 genes upregulated and 1,603 genes downregulated after ANIT administration (Figure 7). In contrast, cis-TSG treatment significantly modulated these gene expressions towards normal levels observed in the control group. The gene coding for sphingomyelin phosphodiesterase 3 (*Smpd3*) was significantly upregulated in the model group compared to the control group. Treatment with *cis*-TSG markedly downregulated *Smpd3* expression, restoring it to levels comparable to the control group (Figure 7C). Similarly, the gene coding for phosphoserine aminotransferase 1 (*Psat1*), a key enzyme in serine biosynthesis pathway, showed a similar expression pattern. These results align with previous findings that elevated ceramide synthesis contributes to liver injury (Morigny et al., 2020). Phospholipid phosphatase 2 (*Plpp2*), an enzyme involved in phosphatidic acid metabolism, was significantly upregulated in the model group. This upregulation was reversed by *cis*-TSG treatment, aligning with the lipidomics data that showed a reduction in phosphatidic acid (PA) levels in the *cis*-TSG-treated group (Li et al., 2006). These transcriptomic findings are consistent with metabolomics and lipidomics analyses, suggesting that *cis*-TSG exerts its hepatoprotective effects by modulating key genes involved in lipid metabolism. The KEGG pathway enrichment analysis further supported these results, highlighting the significant impact of lipid metabolism pathways in the protective effects of *cis*-TSG (Figure 7D). Overall, these results indicate that *cis*-TSG modulates critical genes involved in lipid and bile acid metabolism, providing a mechanistic basis for its hepatoprotective effects in ANIT-induced cholestasis.

### 3.8 *cis-TSG prevents liver lipotoxicity via* FFA-induced PPARα activation

**FIGURE 8:**
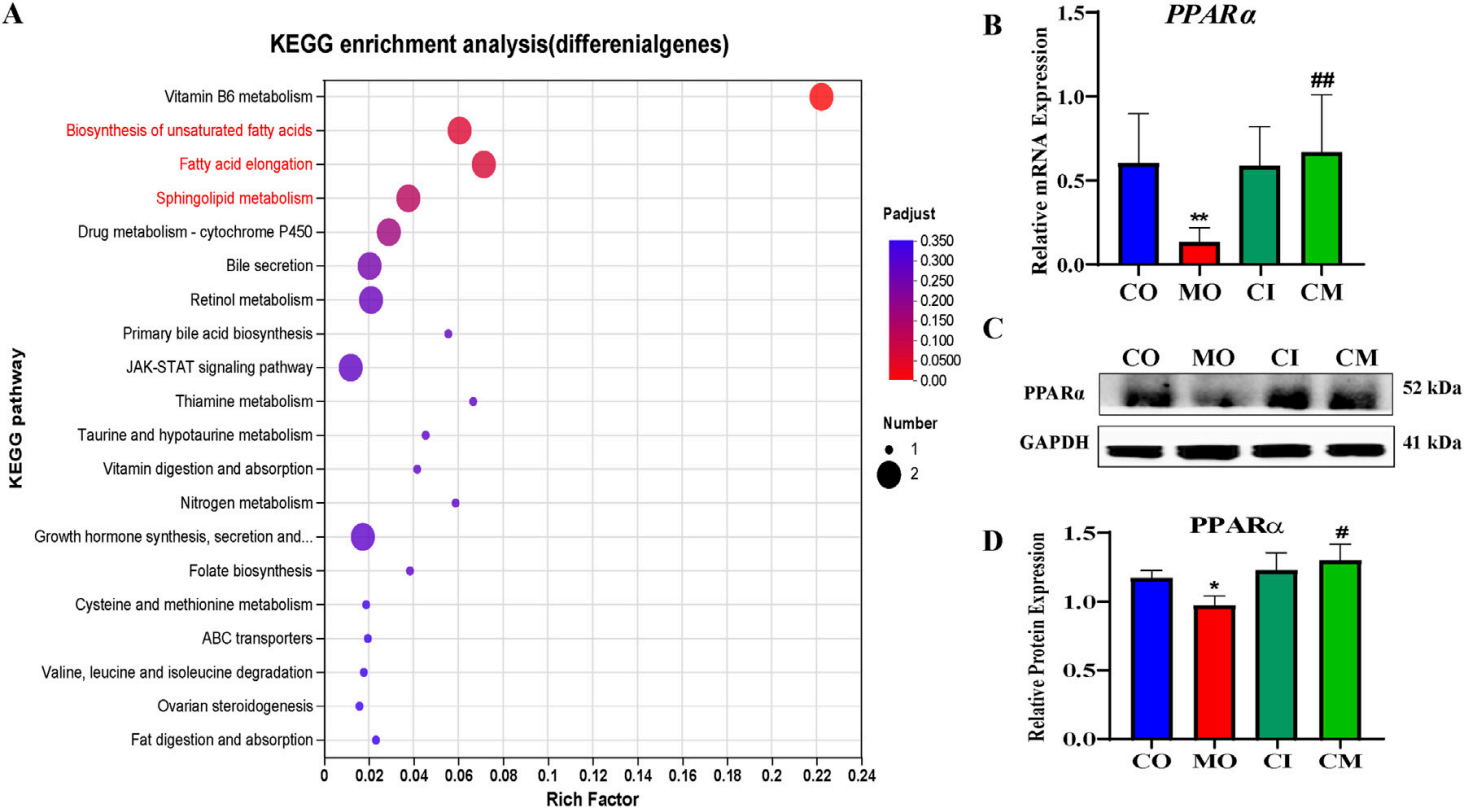
PPARα is important in cis-TSG treatment of lipotoxicity in cholestasis mouse. A KEGG enrichment analysis of deferentially expressed genes in CM vs MO B RT-PCR showing the mRNA expression level of PPARα between the four groups.C western blot showing the protein expression of PPARα between the four groups.CO, control group; CI, cis-TSG only group; CM, cis-TSG + ANIT group; MO, ANITgroup.

Based on earlier findings, it is believed that PPARα increases the production of MUFA by influencing the mRNA levels of genes responsible for fatty acid oxidation, synthesis, transport, and the formation of triacylglycerol (TAGs) (Tian et al., 2020). As a result, we theorized that the higher concentrations of MUFAs and TAGs in the group treated with *cis*-TSG, compared to the model group, could be attributed to the further activation of the PPARα pathway by *cis*-TSG. To test our hypothesis, we investigated the mRNA and protein expression levels of PPAR*α*. As presented in Figure 8, RT-qPCR and western blot analyses confirmed a significant reduction in the mRNA and protein expression levels of PPAR*α* in liver cells of the model group compared to the control group. However, treatment with *cis*-TSG notably reversed this reduction, restoring PPAR*α* expression to levels comparable to the control group. These findings support our hypothesis that *cis*-TSG mitigates liver lipotoxicity through FFA-induced activation of PPAR*α*. This activation likely contributes to the observed hepatoprotective effects in the *cis*-TSG-treated group.

## 4 Discussion

Available evidence suggests that PM has both protective and potentially harmful effects on the liver (Xue et al., 2020; Ruan et al., 2019; Lee et al., 2012). However, current research has not yet identified the specific compounds or explained the underlying mechanism responsible for PM’s dual effects on liver health. Therefore, it is important to conduct detailed investigations into the effects of PM on liver health, especially considering its widespread use in clinics for treating liver disorders (Xue et al., 2020). PM contains several natural compounds, and the stereo-ismeric stilbene glycoside, TSG, has been identified as one of the main bioactive compounds responsible for most of PM’s pharmacological and toxicological effects (Xue et al., 2020; Yu et al., 2020; Liu et al., 2024a). Thus, in this study, we provide evidence of the pharmacological effect and mechanism of action of *cis*-TSG against ANIT-induced cholestasis model in C57BL/6J male mice.

ANIT-induced intrahepatic cholestasis disrupts bile formation, secretion, and excretion, leading to lipid homeostasis disruption and oxidative stress, which subsequently triggers liver injury that impacts liver function (Li et al., 2022). The release of liver-specific enzymes, such as ALT and AST, into the bloodstream serves as an indirect assessment of liver function. Elevated levels of these enzymes indicate liver injury (Liu et al., 2022; Zhang C. et al., 2021). Treatment with *cis*-TSG significantly reduced ALT and AST levels, demonstrating a potential hepatoprotective effect. Our findings also showed that *cis*-TSG significantly reduces liver tissue injury. This was demonstrated by an improvement in liver tissue structure, with minimal inflammatory cell infiltration and hepatocyte necrosis. Additionally, *cis*-TSG restored disrupted bile formation, secretion, and excretion.

Previous studies have demonstrated that several metabolic pathways related to lipids and bile acids are disrupted during liver disorders. In fact, in chemical-induced cholestasis, several transporters and enzymes essential in bile acid biosynthesis and bilirubin excretion are mostly impaired (Paul et al., 2022). Consistent with our expectations, we found that *cis*-TSG treatment potentially regulates several of these metabolic pathways, such as bile acid biosynthesis, glycerophospholipid metabolism, and sphingolipid metabolism. PUFAs are known for their regulatory roles in energy metabolism and their anti-inflammatory properties, reducing the risk of metabolic diseases such as hyperlipidemia, cardiovascular disease, type 2 diabetes, and cancer (Albracht-Schulte et al., 2018; Liu et al., 2021). Also in agreement with previous findings [ref], we also found that in the ANIT-induced cholestasis model, several PUFAs such as hexadecadienoic acid (FFA, 16:2), α-linolenic acid (FFA, 18:3, ω-3), stearidonic acid (FFA 18:4, ω-3), eicosadienoic acid (FFA 20:2, ω-6), and mead acid (FFA 20:3, ω-9) concentration levels were drastically reduced. Interestingly, however, treatment with *cis*-TSG increased the concentration level of these PUFAs comparable to their normal level. The analysis of differential lipid metabolites also indicates a significant reduction in MUFAs such as oleic acid, myristoleic acid, and palmitoleic acid in the chemical-induced cholestatic model. Comparable to PUFAs, MUFAs, including oleic acid, possess antioxidant and anti-inflammatory properties, and notably serve as potent ligands of PPARα (Liu et al., 2024b). The *cis*-TSG treatment effectively increased MUFA levels, indicating its potential influence on PPARα and its associated pathways.

A substantial body of research indicates that sphingolipids, notably ceramides, play a significant role in the pathogenesis of several metabolic diseases (Choi et al., 2021). Ceramides are synthesized via a ubiquitous pathway initiated by the condensation of palmitoyl-CoA, an amino acid (usually serine), and a variable fatty acid (Choi et al., 2021), and they are known to inhibit mitochondrial electron transport and fatty acid β-oxidation, leading to disturbances in lipid homeostasis and lipotoxicity (Choi et al., 2021). Here, in our ANIT-induced cholestatic model, we detected notable changes in plasma and liver lipid profiles, as well as alterations in liver lipid-related gene expressions. Sphingomyelins (SMs) are enzymatically converted into ceramides by sphingomyelin phosphodiesterases (SMPDs), while phospholipid phosphatases (PLPPs) convert DAGs to PAs, which then yield PCs, the precursors of SMs. In our study, livers of ANIT-induced cholestatic mice exhibited heightened ceramide levels and increased hepatic expression of Smpd3 and plpp2, both crucial in the progression of liver injury (Fu et al., 2019). Furthermore, we observed elevated hepatic expression of psat1, the key enzyme in the serine synthesis pathway (Luo et al., 2022). Prior findings have shown that supplementation with PUFAs diminishes ceramide levels, thereby restoring healthy mitochondrial homeostasis (Chen et al., 2024). Our investigation revealed that mouse livers treated with *cis*-TSG displayed a significant increase in levels of MUFAs and PUFAs, alongside heightened expression of elovl3. This redirection of the ceramide synthesis pathway towards the biosynthesis of polyunsaturated fatty acids substantially ameliorated lipid homeostasis disturbance, reduced lipotoxicity, and ultimately restored normal health in the mice.

The literature suggests that FMO3 may contribute to an increase in hyperlipidemia (lipogenesis) by converting TMA to TMAO. Thus, it inhibits the rate-limiting step in the biosynthesis pathway of bile acids from cholesterol, which is controlled by CYP7A1 and CYP27A1. This alteration in cholesterol metabolism leads to the production of lipids, resulting in dyslipidemia in the liver (Canyelles et al., 2018). Our transcriptomic data reveals that while FMO3 was upregulated in the model group, cis-TSG downregulated this enzyme, indicating a potential restorative effect in cholestatic dyslipidemia. In hepatocytes, canalicular ABC (ATP Binding Cassette) transporters, including ABCC2/MRP2, ABCG2/BCRP, ABCB1/MDR1/P-glycoprotein, and ABCB11/BSEP (bile salt export pump), are crucial for expelling endogenous and exogenous substances into the bile (Dikkers and Tietge, 2010; Ambrus et al., 2021; Sundaram and Björnsson, 2017). Conversely, basolateral or sinusoidal ABC transporters, such as ABCC3/MRP3 and ABCC4/MRP4, facilitate the removal of toxic compounds into the venous blood (Ambrus et al., 2021). These transporters collectively play a crucial role in protecting hepatocytes from the excessive accumulation of toxins, with ABCB1 (P-glycoprotein) being the most extensively studied. It functions as a biological barrier by actively transporting toxic agents, including bile acids, out of cells. Both laboratory and clinical studies have demonstrated the pivotal role of P-glycoprotein in regulating drug absorption and distribution (Lin and Yamazaki, 2003). In our study, the expression of abcb1a was significantly upregulated in the model group, signifying elevated accumulation of bile acids in the liver. Conversely, in the CM group, the expression of abcb1a was downregulated, resulting in a reduction in the accumulated bile acids and the alleviation of cholestatic liver injury by *cis*-TSG.

The activation of PPARα induces alterations in intracellular fatty acid metabolism, leading to the oxidation or sequestration of saturated fatty acids (SFAs) and a reduction in lipotoxicity (Nolan and Larter, 2009). Fatty acid metabolism and ketogenesis are recognized as the most evolutionarily conserved PPARα-regulated biological processes between humans and mice (Pawlak et al., 2015). In the liver, energy metabolism is governed by the mitochondrial and peroxisomal fatty acid beta-oxidation pathways, as well as the microsomal omega-oxidation pathway, all of which are under the regulatory control of the PPARα. Functioning as a receptor for peroxisome proliferators, PPARα serves as a pivotal sensor for fatty acids, thereby playing a critical role in lipid metabolism (Reddy and Rao, 2006).

Our major finding shows that elevated levels of FFA, specifically MUFA and PUFA, are due to the fact that *cis*-TSG treatment induces the expression levels of PPARα. This activity by *cis*-TSG enhances beta-oxidation, promoting a healthy energy equilibrium. As a result, it effectively addresses dyslipidemia induced by cholestasis and restores normal physiological health in mice. The findings also strongly suggest that FFA-induced PPARα activation (agonism) may be a fundamental mechanism contributing to the liver-protective effects of *cis*-TSG against ANIT-induced cholestasis. This is particularly noteworthy as synthetic fibrates, which are PPARα agonists like fenofibrate, ciprofibrate, and gemfibrozil, are commonly used in clinical settings for addressing lipotoxicity (Pawlak et al., 2015).

Within healthy tissues, when caloric intake surpasses energy demand, surplus free fatty acids are processed into inert triglycerides and stored within lipid droplets in cells (Choi et al., 2021). Our findings indicate that the administration of cis-TSG facilitates the production of diacylglycerols (DAGs) and triacylglycerols (TAGs), which synergize with the activation of the beta-oxidation pathway, showcasing the robust liver-protective properties of cis-TSG. Additionally, it is known that MUFA triggers the synthesis of phosphatidylinositols (PIs), which are minor components of phospholipids but play a crucial role in stress reduction and mitigating cell death. We observed a significant increase in PI and lysophosphatidylinositol (LPI) levels in the *cis*-TSG-treated group compared to the control, underscoring the liver-protective effects of cis-TSG by mitigating oxidative and organ stress (Thürmer et al., 2022). In conclusion, our study proves that the stereo-isomeric stilbene glycoside *cis*-TSG from PM exhibits significant hepatoprotective effects in an ANIT-induced cholestatic mouse model. *Cis-TSG* reduced ANIT-induced lipotoxicity by modulating the lipid metabolism pathway, specifically through MUFA-induced PPARα activation. These findings highlight the potential of *cis*-TSG to alleviate cholestasis and improve liver function.

## Data Availability

The original contributions presented in the study are included in the article/Supplementary Material, further inquiries can be directed to the corresponding authors.

## Glossary

5-OxoETE: 5-Oxo-eicosatetraenoic acid
AhR: Aryl hydrocarbon receptor
ALT: Alanine amino transferase
ANIT: Alpha-naphthyl isothiocyanate
APAP: Acetaminophen
AST: Aspartate amino transferase
BAs: Bile acids
BCA: Bicinchoninic acid
BEH: Ethylene bridged hybrid
BSEP: Bile salt export pump
C57BL/6J: C57 black 6, Jackson Laboratory
**CA-*d***_**4**_: Cholic-2,2,4,4-*d*4 acid
CD: Compound discoverer
cDNA: complementary deoxyribonucleic acid
CMC-Na: Sodium carboxymethyl cellulose
CV-ANOVA: Coefficient of variance-Analysis of variance
CYP2B10: Cytochrome P450 family 2 subfamily B polypeptide 1o
CYP3A11: Cytochrome P450 family 3 subfamily A member 11
CYP7A1: Cytochrome P450 family 7 subfamily A member 1
CYP8B1: Cytochrome P450 family 8 subfamily B member 1
DAD: Diode-array detection
ddH2O: Double-distilled water
DEPC: Diethyl pyrocarbonate
DHOME: Dihydroxy-octadecenoic acid
DI: Distal ileum
ECL: Chemiluminescence
FXR: Farnesoid X receptor
GAPDH: Glyceraldehyde-3-phosphate dehydrogenase
H-E: Hematoxylin-eosin
HESI: Heated electrospray ionization
HMDB: Human metabolome database
HO-1: Heme oxygenase 1
HPLC: High-performance liquid chromatography
HSS T3: High strength silica T3 (C18 -trifunctionally bonded)
IC: Intrahepatic cholestasis
IgG: Immunoglobulin G
IL-6: Interleukin-6
KEGG: Kyoto encyclopedia genes and genomes
LC-MS: Liquid chromatography-mass spectrometry
LPC: Lysophosphatidylcholine
LysoPC: Lysophosphatidylcholine
MDR2: Multidrug resistance 2
METLIN: Metabolite mass spectral database
mRNA: messenger RNA
NCE: Normalized collision energy
NF-kB: Nuclear factor kappa B
NIH: National institute of health
NMR: Nuclear magnetic Resonance
Nrf2: Nuclear factor erythroid 2-related factor 2
NTCP: Sodium taurocholate co-transporting polypeptide (NTCP)
OPLS-DA: Orthogonal partial least-squares discriminant analysis
PCA: Principal Component analysis
PM: *Polygonum multiflorum*
PPARα: Peroxisome proliferator-activated receptor alpha
PVDF: Polyvinylidene fluoride
PXR: Pregnane X receptor
QC: Quality control
RNA: Ribonucleic acid
ROS: Reactive oxygen species
RT-qPCR: Real time quantitative Polymerase chain reaction
SD: Standard deviation
SDS-PAGE: Sodium dodecylsulphate polyacrylamide gel electrophoresis
SIMCA: Soft independent modelling by class analogy
SULT2A1: Sulfotransferase family 2A member 1
TSG: 2,3,5,4′-tetrahydroxystilbene-2-*O*-*β*-D-glucopyranoside
UGT1A1: Uridine 5′-diphospho-glucuronosyltransferase Family 1 Member A1
UGT1B1: Uridine 5′-diphospho-glucuronosyltransferase Family 1 polypeptide B1
UGT2B4: Uridine 5′-diphospho-glucuronosyltransferase Family 1 member B4
UHPLC: Ultra-high performance liquid chromatography
UHPLC-MS/MS: Ultra-high performance liquid chromatography-tandem mass spectrometry
UV: Ultra-violet
VIP: Variable importance in the projection
