# Supplementary material for: Integrated multi-omics analyses combined with western blotting discovered that cis-TSG alleviated liver injury via modulating lipid metabolism
Source: Front Pharmacol. 2024 Nov 20;15:1485035. doi: 10.3389/fphar.2024.1485035 (PMC11614611; doi:10.3389/fphar.2024.1485035)
Supplement: Supplementary file 1 [file Table1.docx]

| **Gene** | **Forward Primer (5' to 3' sequence)** | **Reverse Primer (5' to 3' sequence)** |
| --- | --- | --- |
| CYP7A1/CYPVII; CYPVIIc/ | GAAGGCTAAGACGCACCTCG | CTTGGCCAGCACTCTGTAATGC |
| PPARα | AACTGACGTTTGTGGCTGGT | GCTCTCTGTGTCCACCATGT |
