# Supplementary material for: Integrated multi-omics analyses combined with western blotting discovered that cis-TSG alleviated liver injury via modulating lipid metabolism
Source: Front Pharmacol. 2024 Nov 20;15:1485035. doi: 10.3389/fphar.2024.1485035 (PMC11614611; doi:10.3389/fphar.2024.1485035)
Supplement: Supplementary file 2 [file Image1.pdf]

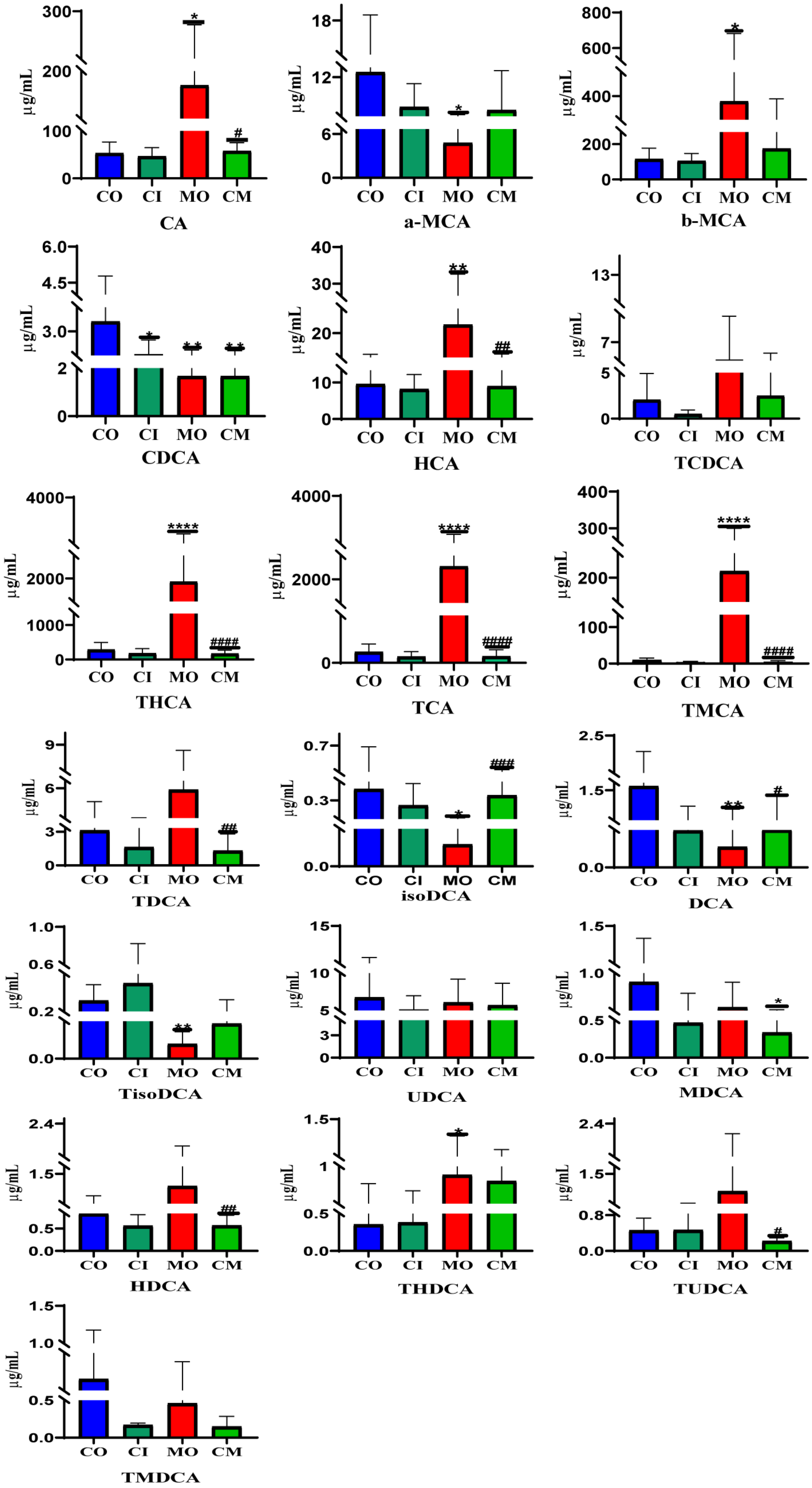

µg/mL
CO CI MO CM
CA
a-MCA
b-MCA
CDCA
HCA
TCDCA
THCA
TCA
TMCA
TDCA
isoDCA
DCA
TisoDCA
UDCA
MDCA
HDCA
THDCA
TUDCA
TMDCA

Fig. S1. Content comparison of individual primary and secondary bile acids in liver.

CA, Cholic acid; a-MCA, alpha-muricholic acid; b-MCA, beta-muricholic acid; CDCA, Chenodeoxycholic acid; HCA, Hyocholic acid; TCDCA, Taurochenodeoxycholic acid; THCA, Taurohyocholic acid/Tauro-γ-muricholic Acid; TCA, Taurocholic acid;HDCA, Hyodeoxycholic acid; TMCA, Tauro-beta-muricholic acid; TDCA, Taurodeoxycholic acid; isoDCA/3-DCA, isodeoxycholic acid (3-deoxycholic acid); DCA, Deoxycholic acid;TisoDCA, Tauroisodeoxycholic acid (3β,12α-dihydroxy-5β-cholan-24-oic acid); UDCA, Ursodeoxycholic acid; MDCA, Murideoxycholic acid; HDCA, Hyodeocholic acid; THDCA, Tauroursodeoxycholic acid; TUDCA, Taurohyodeoxycholic acid; Taurohyodeoxycholic acid; MDCA, Tauromurideoxycholic acid.
